# President’s Address

**Published:** 1892-04

**Authors:** L. E. Custer

**Affiliations:** Dayton, O.


### President's Address.

L. E. CUSTER, D.D.S., DAYTON, O.

Read before the Mississippi Valley Dental Association March 9, 1892.

This society meets to-day for the forty-ninth time. Properly speaking this is the forty-eighth annual session, and two years hence will have rounded out half a century of usefulness of one of the oldest dental societies in existence.

National, State and local societies have sprung up in her territory and divided the interest, but she still exists, and on account of her age, the good she has done, and the good she is still doing, she will live for years to come.

It is unnecessary to recall her history, and the few words in the way of an address will be concerning the fiftieth anniversary of the society in 1894.

There is a general impression that it should be celebrated in a befitting manner. We would not expect this to be a national meeting, but one characterized by a large attendance of dentists from the original territory comprised by the society, and the attendance of many of those who were once members, but have since become associated elsewhere. Some may question so early an agitation of this movement, but the reader having just participated in the preparations for this meeting has a vivid impression of the importance of an early beginning in the preparations for any dental meeting.

These preparations should be begun early for many reasons. There is a peculiar interest and fascination shown where large numbers of people come together. The farmer attends the fair and circus as much for the bustle as for the peanuts and lemonade.. We instinctively "follow the crowd," and when we are assured there will be a big dental meeting we always attend.

In order to invite attendance, the program must not only be good, but must be announced beforehand, to do which requires time. The movement of all committees is slow, and it requires time to arrange a definite plan of action. It requires time to secure good papers, and it is always difficult to obtain clinics and other attractive features. A good paper cannot be prepared in a day or a week, and it requires time to perfect an appliance. Many men have ideas and crude appliances, which may be completed for a coming meeting.

A society cannot expect to be well attended which sends out its program a week before the time. To have a meeting and program announced a year previous indicates that the preparations have not only been perfected, but that each one who participates will come well prepared.

Besides the early preparation of a program for any dental meeting, the proposed anniversary of this society comes at a time when the interest will be divided with another important meeting—the Columbian—and it behooves us to early look to the interests of this society. I do not mean to detract from the Columbian Dental Congress, for we are all interested in its success, but on account of that meeting it will be more difficult to secure a good program, and so if this is to be a success we must be at work. Let the interest in this society increase. Let next year's meeting be a better one than this year's, and awaken a general interest in preparation for the anniversary.

We should begin the work for another reason. There is no doubt but that the World's Columbian Dental Meeting will be the best meeting we will have the opportunity of attending for some time, and the success of that meeting will make it all the more difficult to secure a good meeting of the Mississippi Valley Dental Society. The brilliancy of the Columbian meeting will outshine any similar effort following in so short a time. If it were possible it would be well for this society to take an active part in the Columbian meeting by way of presenting statistics, the literature which has developed from it, models of the most important appliances which were first shown here, and all the marked improvements in dentistry which emanated from this society.

A few suggestions might be offered regarding the anniversary of this society itself, if it be decided to have such.

Let the presiding officer be one of the few patriarchs remaining whose very name will insure success. Let there be a short history gleaned from the minutes of the work done, a short report of the literature, a similar report of instruments and methods presented here. Then a program of new papers, and by all means a good number of clinics. These papers may be presented by men of known reputation, and from a distance, and the discussions opened by competent persons, if we find it to be a success in this meeting, which we will now proceed to.

---

DISCUSSION ON PRESIDENT'S ADDRESS.

*Dr. J. Taft.*—I think that the paper or address read by Dr. Custer, our President, should be discussed, as there are many points of interest in it.

Dr. M. H. Fletcher then spoke of the Columbian meeting, and of the object of the committee, which seems to be to gather together some of the points spoken of by the President, he then read the list of questions sent out to be answered by the State Committees for the Columbian Dental Congress.

*Dr. J. Taft.*—I might refer to what has been done in this work, the committee was appointed one year and a half ago, for the purpose of enlisting the interest of the profession throughout the United States, and obtaining a promise from them to attend that meeting and do whatever each could for its interest. A committee has been organized in every State and Territory in the Union, with perhaps one or two exceptions, in which I presume the persons addressed were absent, for this purpose. The question very naturally arose, what shall this committee do? The questions indicate the line of work in which these committees are at work. It is gratifying to know there is emulation among these committees. The object is two-fold. One is to arrive at the present status of the profession, if these questions are answered correctly, it will give us the correct data. The other is, it will give us the opportunity to gather together material for a History of Dentistry throughout the country better than has ever been before. The opportunity now occurs, and it is well that it should be taken advantage of. The question was then asked: "What shall be done with all this mass of material when brought together?" These committees consist of from four to seven persons. The Chairman of each committee assigns a certain district to each of the members, which he is to work up, and in which he is to ascertain the facts that will answer these questions, that is to be sent into the Secretary of the Committee. This you see, will bring together a large mass of material, so that it will not be long before matter will have accumulated which will afford material for the preparation of a History of Dentistry superior to anything heretofore obtained. At the last meeting in Chicago, a committee was appointed to revise and bring it together in the proper form for publication. A synopsis will be given at the Columbian Dental Congress, which will give an account of the present status.

*Dr. Fletcher.*—There is material here in the records of this Society for a very interesting report, this being the oldest Society in operation in this part of the country.

*Dr. C. M. Wright.*—If I understand the address of the President correctly, it was that we, as an individual society should do something—have a small fireworks of our own— to celebrate our semi-centennial anniversary; while this might be a good plan for the Columbian meeting, which we are all interested in, it seems necessary that we should do something for our own meeting.

*Dr. Fletcher.*—I fully coincide with the suggestions of the President, as well as those of Dr. Wright.

*Dr. H. A. Smith.*—Dr. Fletcher's remarks that we have in this town some very old literature relating to the formation of this Society, I fully agree with, there are some very interesting facts connected with the organization of this Society.

*Dr. Jay.*—I have some very old literature in regard to the formation of Dental Associations, I do not know if I have anything in regard to the Mississippi Valley Association, but if I have, will be glad to contribute it.

Dr. Fletcher moved that a committee be appointed to take this matter in hand and gather all the material possible to make this history complete. Committee appointed: Drs. Fletcher, Taft and Wright.

